# Acute cataract by a high-intensity focused ultrasound procedure: a case report

**DOI:** 10.1186/s12886-022-02390-2

**Published:** 2022-04-09

**Authors:** Toru Ikoma, Teppei Shibata, Naoko Shibata, Tsuyoshi Mito, Eri Kubo, Hiroshi Sasaki

**Affiliations:** Department of Ophthalmology, Kanazawa Medical University, 1-1 Daigaku, Uchinada, Kahoku, Ishikawa 920-0293 Japan

**Keywords:** Intense focused ultrasound procedure, Drop-like dense lens opacities, Rosette cataract, Case report

## Abstract

**Background:**

We report a case of acute onset of cataract after eyelid rejuvenation tightening with intense focused ultrasound (IFUS) treatment without using a protection device.

**Case presentation:**

A 47-year-old female patient presented at the outpatient clinic with blurred vision in her left eye immediately after undergoing an eyelid tightening procedure, using IFUS, seven days prior. The patient had decreased vision in her left eye, caused by an acute cataract with several drop-like opacities and a rosette-like posterior subcapsular cataract. One month after her first visit, the patient’s visual acuity in her left eye decreased to 20/630. A Swept-Source Anterior Segment optical coherence tomography confirmed that the posterior capsule was not ruptured. The patient underwent uneventful phacoemulsification cataract surgery with intraocular lens implantation, which resulted in full visual recovery.

**Conclusions:**

This case emphasized the need to evaluate possible ocular side effects, resulting from periocular IFUS without a protection device, including severe cataract requiring surgery.

**Supplementary Information:**

The online version contains supplementary material available at 10.1186/s12886-022-02390-2.

## Background

High-intensity focused ultrasound (HIFU) uses ultrasound waves to achieve therapeutic effects by destroying deep-localized tissue. It has been previously indicated as a treatment for prostate cancer [1] and has recently been suggested as an alternative treatment for glaucoma [2]. HIFU is also used as a cosmetic treatment, inducing selective thermal coagulation and delivering heat to the dermis and subdermis, including the superficial musculoaponeurotic system, to improve sagging skin and to increase collagen through intense focused ultrasound (IFUS) treatment [3]. Recently, IFUS has been performed as a "non-cutting eye treatment," consisting of the irradiation of the periocular area of sagging eyelids. In this treatment, the medical contact eye protection must be used. We report a case of acute progressive cataract, following IFUS.

## Case presentation

**Fig. 1:**
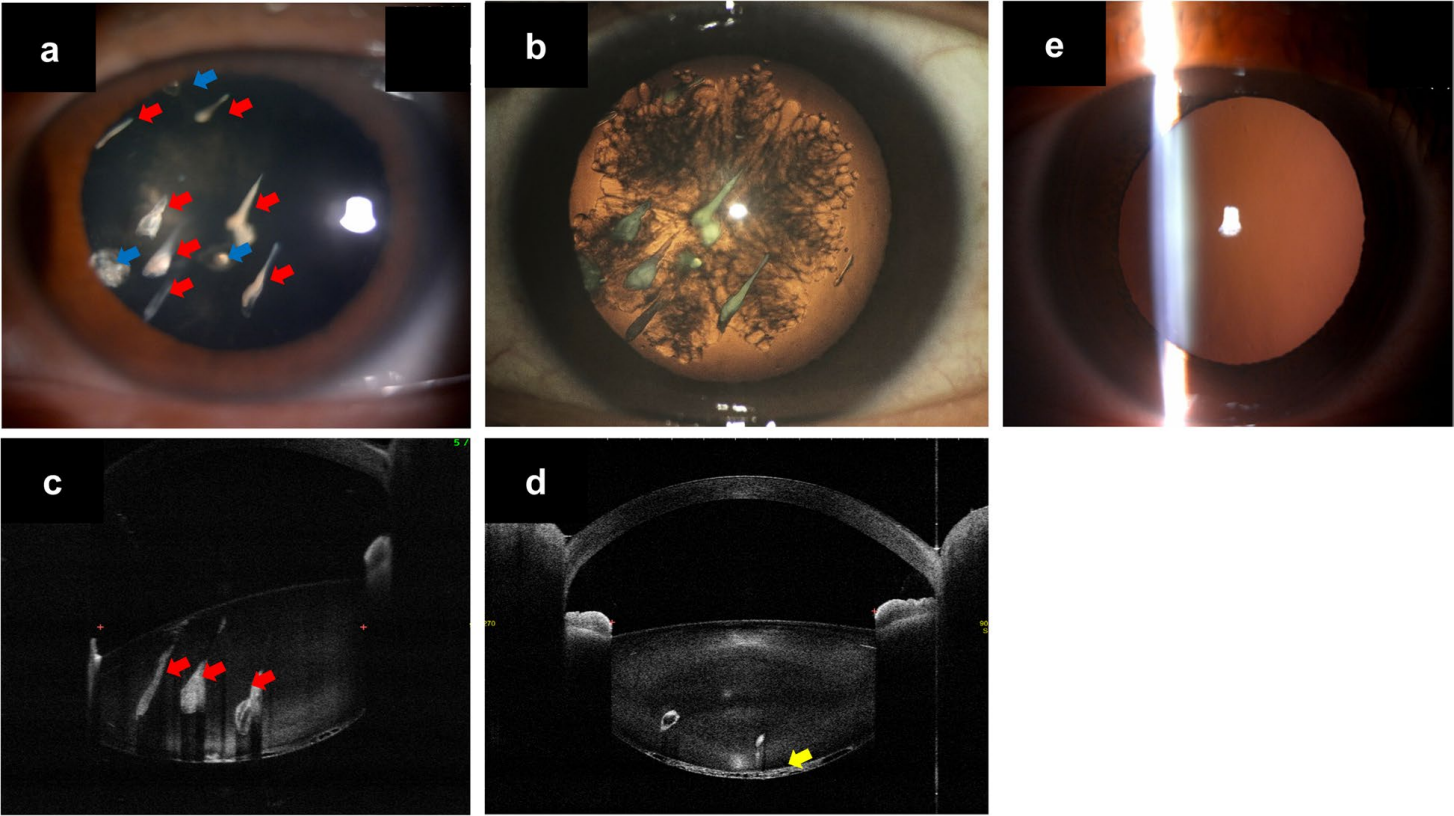
Eyes, seven days after the intense focused ultrasound treatment. Several drop-like opacifications and rosette-like posterior subcapsular cataract (PSC) can be observed in the patient’s left eye, which were documented as acute cataract by slit-lamp photography. **a** Seven drop-like dense lens opacities were aligned and obliquely localized from the center of the lens to the perinucleus region (red arrows). Three lens sense opacities extended vertically to the posterior capsule (blue arrows) by slit-lamp photography. **b** Retro-illumination image of the patient’s left eye by a Casey Eye Institute camera system [4]. Rosette-shape PSC was observed. **c d** Anterior optical coherence tomography showing drop-like opacity (red arrows) in the patient’s lens (**c**) and PSC (yellow arrow) (**d**); the patient’s posterior capsule did not appear to be ruptured. **e** No lens opacity was observed in the patient’s right eye

A 47-year-old female patient consulted our outpatient clinic with blurred vision in her left eye, immediately after undergoing IFUS assisted-facelifting (irradiation dose unknown). She reported that a beautician treated her using IFUS to reduce eyelid laxity seven days prior. The patient was not aware of any difference in the duration or intensity of the IFUS procedure between her left and right eyes. However, as a protective eye shield was not used, she became aware of a decrease in vision in her left eye following the procedure. Aside from myopia, she had an unremarkable medical and ocular history. There was no history of ocular trauma, diabetes, corticosteroid use, or other systemic diseases or drug use. She had an uncorrected distance visual acuity of 20/20 in her right eye and 20/1000 in her left eye. There were no abnormalities in the patient’s conjunctiva, cornea, and iris. Her light reflexes were intact, while her intraocular pressure values were normal. For her left eye, seven drop-like dense opacities, aligned horizontally, and three lens opacities, extending vertically, anterior to her posterior capsule, were noted (Fig. 1a and b). The patient’s posterior subcapsular cataract (PSC) had a rosette shape; therefore, it was identified as a rosette cataract (Fig. 1b). While a Swept-Source Anterior Segment optical coherence tomography (SS-ASOCT) showed drop-like opacities in the patient’s lens (Fig. 1c) and PSC (Fig. 1d), her posterior capsule was not ruptured (Fig. 1c and d). Fundus photographs were not taken due to severe posterior capsule opacity. The macular optical coherence tomography showed no abnormalities (see Additional file 1). In contrast, no lens opacities were noted in her right eye (Fig. 1e).

**Fig. 2:**
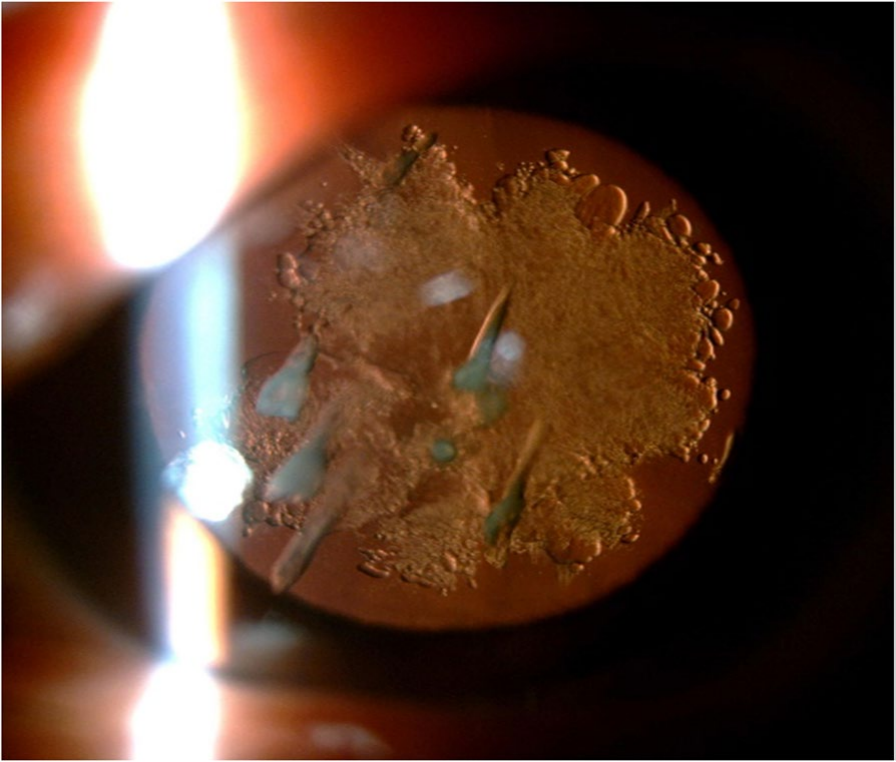
Retro-illumination image of the patient’s left eye by slit-lamp photography, one month after her first visit to the clinic. Several drop-like opacities and rosette-like PSC were still observed and documented as acute cataracts. The area of PSC was slightly smaller than it was on the patient’s first visit

**Fig. 3:**
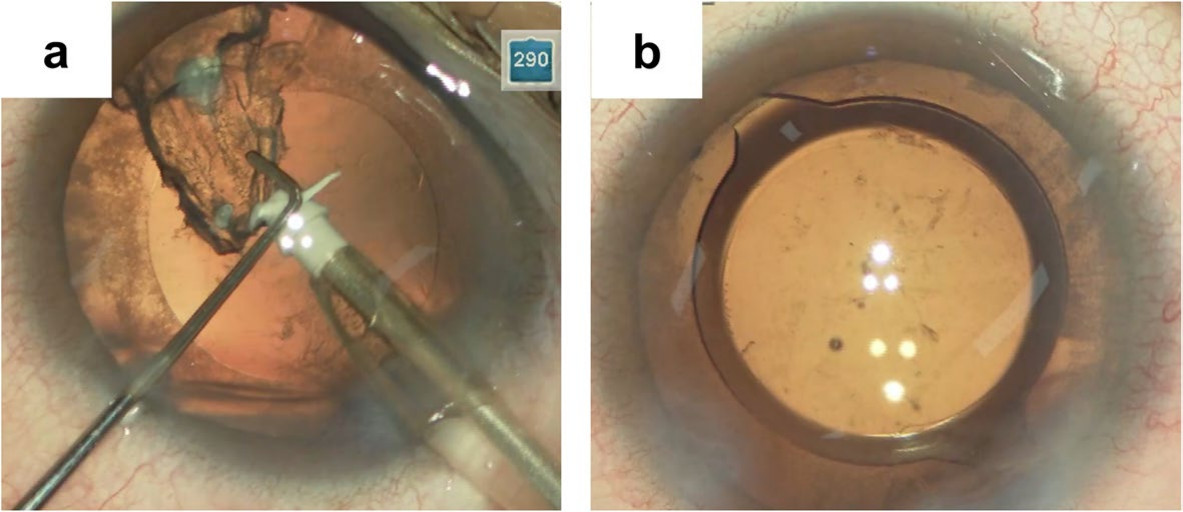
Intra-operative images of the patient’s left eye. **a** After nucleus removal, the cortex containing some of the material of drop-like opacity was safely removed, and the posterior capsule was not ruptured. **b** At the end of the surgery, IOL was implanted in the capsular bag

**Fig. 4:**
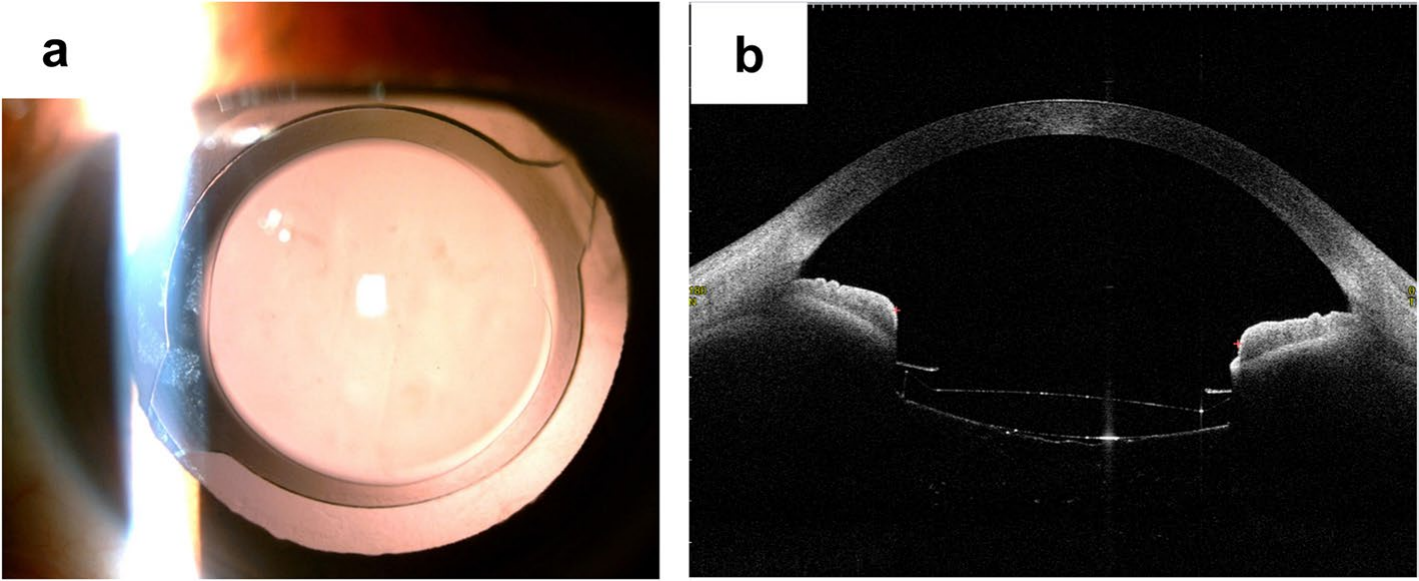
IOL image of the patient’s left eye on the seventh postoperative day. **a b** Retro illumination image by a slit-lamp photograph (**a**); anterior segment image by Swept- Source Anterior Segment optical coherence tomography showing no deviation or tilt in the position of the patient’s intraocular lens

One month after the first visit, the patient returned to the hospital to undergo cataract surgery of her left eye to achieve improved vision. Lens examination of her left eye at that time showed no drop opacity changes, while a slight improvement in her posterior capsule opacity was seen (Fig. 2). However, the corrected distance visual acuity of her left eye was 20/630; therefore, phacoemulsification cataract surgery with implantation of monofocal intraocular lens (IOL) was performed. Given that the patient’s lens was not hard, ultrasonic phacoemulsification was not conducted; irrigation and aspiration were performed instead. The drop-like dense lens opacities were aspirated while pulling the chopper without damaging the patient’s posterior capsule as they were harder than the surrounding lens fibers. The IOL was implanted in the lens capsule bag (Fig. 3). On the seventh postoperative day, the corrected visual acuity of the patient’s left eye improved to 20/20, while SS-ASOCT showed no deviation or tilt in the position of the IOL (Fig. 4).

## Discussion and conclusions

In this report, we described an acute onset of cataract, caused by IFUS performed without protection devices. IFUS reached the lens of the patient’s left eye through her eyelid and caused thermal coagulation of lens proteins, according to the irradiation site. This resulted in drop-like lens opacities and posterior rosette cataracts. In our case, the patient required cataract surgery due to severe vision loss resulting from residual PSC and drop-like lens opacities even after one month. However, no lens opacity was observed in the patient’s right eye. Our patient may have received IFUS from an unlicensed beautician, rather than a certified physician. The differences in the adhesive angle of the probe, duration of irradiation, and distance between the probe and lens may have caused the different incidence of lens opacity between the patient’s left and right lenses. Rosette cataracts are typically seen in patients who have sustained blunt or penetrating ocular trauma. They are rarely observed in cases of electric shocks, such as a lightning injury, infrared energy exposure or Nd-YAG laser [5–8]. The rosette-shaped opacity, a traumatically induced dysfunction of the lens epithelium, reportedly caused reversible pathological changes in the lens, such as superficial cortical lens fiber edema and localized direct damage to the capsule. These result in osmotic imbalance and fluid collection between the lamellae. Restoration of the osmotic balance, formation of the lens fibers, and clearing of vacuoles resulted in the resolution of the lens opacity.

Since the United States Food and Drug Administration’s approval of IFUS for brow lifting (improvement in sagging), it is currently also used for submental and neck lifting, as well as for improving wrinkles in the décolleté. IFUS causes transient and intermittent pain, redness, edema, and purpura during and after irradiation as side effects when applied to the skin. Nevertheless, it has been previously reported as an effective method for the periorbital area and inferior facial region [9]. For this procedure, the medical contact eye shield must be used. However, several case reports on eye damage secondary to IFUS have been reported. These were attributed to the insufficient or improper use of protection devices [10–15]. Thermal coagulation to eye tissues induces cornea and lens opacification. This results in an acute transient increase in intraocular pressure and myopia [10]. A previous study indicated HIFU keratoplasty as a method to increase corneal curvature, without reporting lens opacity[16]. The treatment did not involve direct contact between the ultrasound transducer and corneal surface, and the ultrasonic coupling agent acted as the protection shield. Duration of irradiation and distance between the probe may be important to avoid lens damage.

While HIFU devices have been recently sold for home use, beauty salons now offer self-service IFUS therapy. In our case, IFUS was performed to treat the eyelid; however, an eye shield was not used. As a result, the patient developed cataract as a complication. The general population should be informed of the possible eye damage caused by periocular IFUS procedures. The correct use of eye shields should be encouraged.

## Supplementary Information

**Additional file 1.**

## Data Availability

All data from the case has been included, including images. The datasets analyzed during the current study are available from the corresponding author on reasonable request.

## Abbreviations

HIFU: High-intensity focused ultrasound
IFUS: Intense focused ultrasound
IOL: Intraocular lens
PSC: Posterior subcapsular cataract
SS-ASOCT: Swept-Source Anterior Segment optical coherence tomography
