# Supplementary figures and images for: Acute cataract by a high-intensity focused ultrasound procedure: a case report
Source: BMC Ophthalmol. 2022 Apr 9;22:164. doi: 10.1186/s12886-022-02390-2 (PMC8994384; doi:10.1186/s12886-022-02390-2)

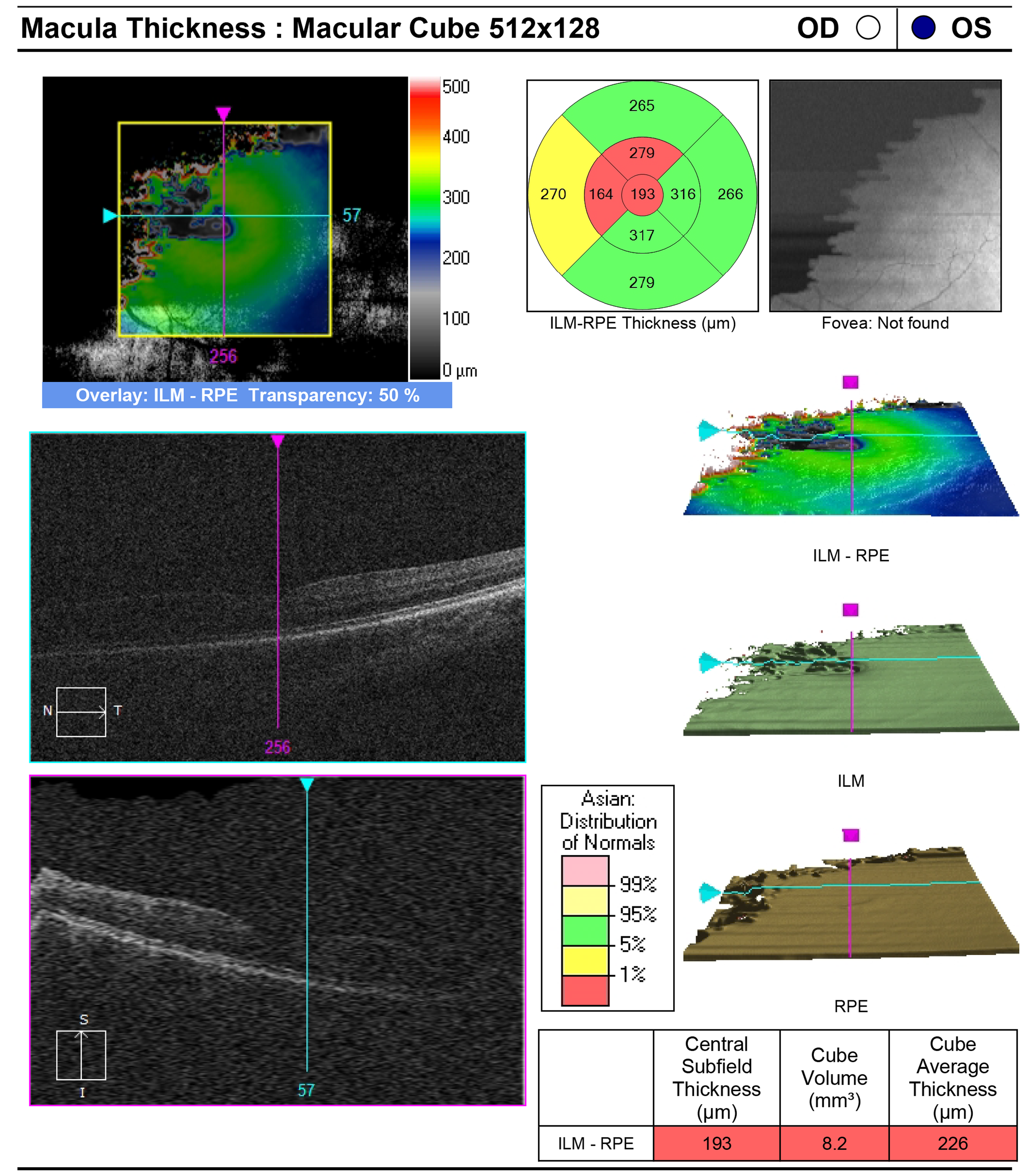

Supplement: Supplementary file 1 — Additional file 1. [file 12886_2022_2390_MOESM1_ESM.tif]
